# Human Costars Family Protein ABRACL Modulates Actin Dynamics and Cell Migration and Associates with Tumorigenic Growth

**DOI:** 10.3390/ijms22042037

**Published:** 2021-02-18

**Authors:** Bo-Yuan Hsiao, Chia-Hsin Chen, Ho-Yi Chi, Pei-Ru Yen, Ying-Zhen Yu, Chia-Hsin Lin, Te-Ling Pang, Wei-Chi Lin, Min-Lun Li, Yi-Chen Yeh, Teh-Ying Chou, Mei-Yu Chen

**Affiliations:** 1 Institute of Biochemistry and Molecular Biology, National Yang-Ming University, Taipei 11221, Taiwan; BYHsiao0916@gmail.com (B.-Y.H.); s7532853@gmail.com (C.-H.C.); joeinter3690@gmail.com (H.-Y.C.); ruby781027@gmail.com (P.-R.Y.); clareyu77@gmail.com (Y.-Z.Y.); celine.pang@uwell.com.tw (T.-L.P.); weichi0905638@gmail.com (W.-C.L.); lazystream@gmail.com (M.-L.L.); tychou@vghtpe.gov.tw (T.-Y.C.); 2 Faculty of Medicine, School of Medicine, National Yang-Ming University, Taipei 11221, Taiwan; chlin1016@cgmh.org.tw; 3 Department of Pathology and Laboratory Medicine, Taipei Veterans General Hospital, Taipei 11217, Taiwan; ycyeh2@vghtpe.gov.tw; 4 Institute of Clinical Medicine, National Yang-Ming University, Taipei 11221, Taiwan; 5 Cancer Progression Research Center, National Yang-Ming University, Taipei 11221, Taiwan

**Keywords:** actin regulator, cell migration, cofilin, colorectal cancer, tumorigenesis

## Abstract

Regulation of cellular actin dynamics is pivotal in driving cell motility. During cancer development, cells migrate to invade and spread; therefore, dysregulation of actin regulators is often associated with cancer progression. Here we report the role of ABRACL, a human homolog of the *Dictyostelium* actin regulator Costars, in migration and tumorigenic growth of cancer cells. We found a correlation between ABRACL expression and the migratory ability of cancer cells. Cell staining revealed the colocalization of ABRACL and F-actin signals at the leading edge of migrating cells. Analysis of the relative F-/G-actin contents in cells lacking or overexpressing ABRACL suggested that ABRACL promotes cellular actin distribution to the polymerized fraction. Physical interaction between ABRACL and cofilin was supported by immunofluorescence staining and proximity ligation. Additionally, ABRACL hindered cofilin-simulated pyrene F-actin fluorescence decay in vitro, indicating a functional interplay. Lastly, analysis on a colorectal cancer cohort demonstrated that high ABRACL expression was associated with distant metastasis, and further exploration showed that depletion of ABRACL expression in colon cancer cells resulted in reduced cell proliferation and tumorigenic growth. Together, results suggest that ABRACL modulates actin dynamics through its interaction with cofilin and thereby regulates cancer cell migration and participates in cancer pathogenesis.

## 1. Introduction

Cell migration is a fundamental mechanism of cancer pathogenesis. For example, in the process of metastasis, various environmental cues direct cancer cells to migrate and invade into surrounding tissues or vessels, causing cancer to spread [1,2,3]. In light of this, therapeutic strategies that target cell migration are potentially effective in curbing cancer progression and improving patient outcomes. To develop these migration-targeting therapeutics, a thorough understanding of the mechanisms underlying cancer cell movements is crucial.

The complexity of cellular events involved in directional cell migration has created challenges toward the full elucidation of the underlying molecular network. The movement of cells in a guided direction requires the coordination of a multitude of cellular machineries to carry out distinct steps: a migrating cell needs to sense the directional cue, transduce the signal into appropriate intracellular activities, establish polarity, and dynamically rearrange the cytoskeletal organization for the generation of motion [4,5]. Much remains to be learned about the molecular linkages between the aforementioned steps and the intricate regulation of the entire cell migration process.

To bridge these knowledge gaps, we have previously performed genetic screening for novel regulators of chemotaxis in the simple eukaryotic model system *Dictyostelium discoideum* and discovered Costars as a regulator of the actin cytoskeleton and cell motility [6]. This ~9 kD protein was designated Costars as it displays significant sequence homology to the C-terminal domain of the actin-binding Rho activating protein (ABRA); ABRA was formerly named the striated muscle activator of Rho signaling (STARS) and is a mammalian actin-binding protein that regulates the serum response factor transcriptional activity through the activation of RhoA [7,8]. *Dictyostelium cosA^-^* cells without the expression of Costars exhibit severely defective chemotactic responses, showing abnormal actin patterns and migrating at a markedly decreased speed, and the cellular F-/G-actin ratios in *cosA^-^* cells are higher than those in wild-type cells [6]. The human Costars homolog ABRACL can functionally replace *Dictyostelium* Costars and rescue the phenotypes of *cosA^-^* cells [6]. Structural analysis of ABRACL has indicated that it is a non-typical winged-helix protein, with a positively charged surface on one side, a negatively charged surface on the other side, and a hydrophobic groove, which may allow ABRACL to interact with other proteins [9]. Despite the revealed structural features, the cellular functions and physiological roles of ABRACL are largely unknown.

In this study, we investigated the role of ABRACL in cancer cell migration. Results demonstrated that the level of ABRACL expression was correlated with the migratory capacity of cells. Data also suggested that ABRACL may regulate cellular actin dynamics, possibly through interacting with the actin regulator cofilin. Furthermore, analyses revealed the upregulation of ABRACL expression in clinical specimens of cancerous tissues and an association of ABRACL expression with proliferation and tumorigenic growth of colorectal cancer cells.

## 2. Results

### 2.1. ABRACL Expression Affects Cancer Cell Migration

**Figure 1:**
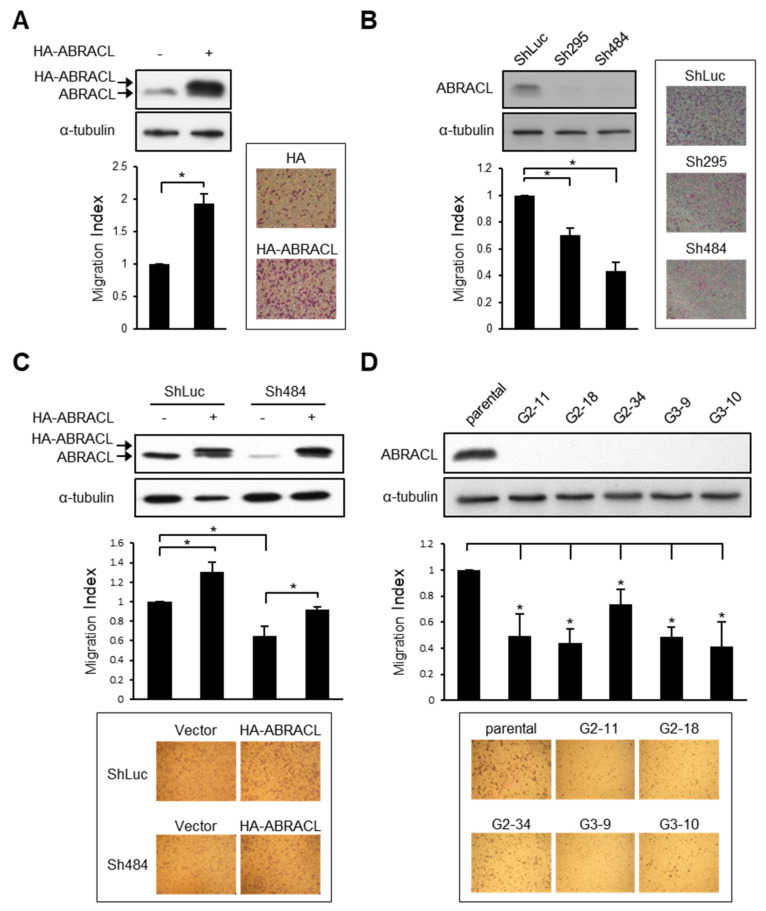
ABRACL expression affects cancer cell migration. (**A**–**D**) Western analysis on cell lysates using antibodies against ABRACL and α-tubulin (loading control), and quantitative results of Transwell migration assays. The migration index was calculated by normalizing the number of migratory cells in each test sample to that of the control; representative micrographs and means ± SD from at least 3 independent experiments are shown; *, *p* < 0.05. (**A**) MDA-MB-231 cells transiently transfected with the control pCMV-HA (−) or pCMV-HA-ABRACL (+) vector. (**B**) Stable clones of HCT116 infected with the control luciferase-targeting (ShLuc) or *ABRACL*-targeting (Sh295 and Sh484) shRNA-expressing lentivirus. (**C**) Stable control (ShLuc) and *ABRACL*-knockdown (Sh484) HCT116 clones transfected with the control HA- (−) or HA-ABRACL (+)-expressing vector. (**D**) Parental and independent *ABRACL*-knockout HCT116 clones generated by CRISPR/Cas9-mediated gene editing using two different *ABRACL*-targeting sequences (G2 and G3).

Given that ABRACL and *Dictyostelium* Costars share remarkable sequence similarity and that ABRACL can rescue the migration defect of Costars-deficient *Dictyostelium* cells [6], we set out to test the role of ABRACL in cancer cell migration. We first profiled ABRACL expression in a panel of human cancer cell lines and found high expressers, such as HCT116, medium expressers, such as MDA-MB-231, and low expressers, such as SW620 (Figure S1). Based on the profiling, we chose to transfect MDA-MBA-231 cells with an ABRACL-expressing vector and perform knockdown or knockout of *ABRACL* expression in HCT116 cells. The resulting sets of isogenic cells expressing ABRACL at different levels were assessed for their ability to migrate. We found that MDA-MB-231 breast cancer cells with transient overexpression of HA-ABRACL showed increased migration as compared to that of control cells in the Transwell assay (Figure 1A). On the other hand, stable clones of HCT116 colon cancer cells infected with two different *ABRACL*-targeting shRNA-expressing lentiviruses (Sh295 and Sh484) showed lower ABRACL expression and exhibited significantly reduced migration as compared to that of the knockdown control (shLuc) cells (Figure 1B). Expressing HA-ABRACL from an RNAi-resistant vector in *ABRACL*-knockdown cells enhanced migratory ability, confirming that the decrease of migration in these knockdown cells was due to the reduction of ABRACL expression (Figure 1C). We also carried out CRISPR/Cas9-mediated genomic editing to obtain *ABRACL*-knockout cells. Two different sequences were used to prepare *ABRACL*-targeting guide RNA (gRNA)-expressing vectors for co-transfection with a Cas9-expressing vector into HCT116 cells. We identified candidate knockout clones by Western analysis and verified the editing at the *ABRACL* locus in these clones by genomic DNA sequencing. All five *ABRACL*-knockout clones examined displayed significantly reduced migration in the Transwell assay (Figure 1D).

**Figure 2:**
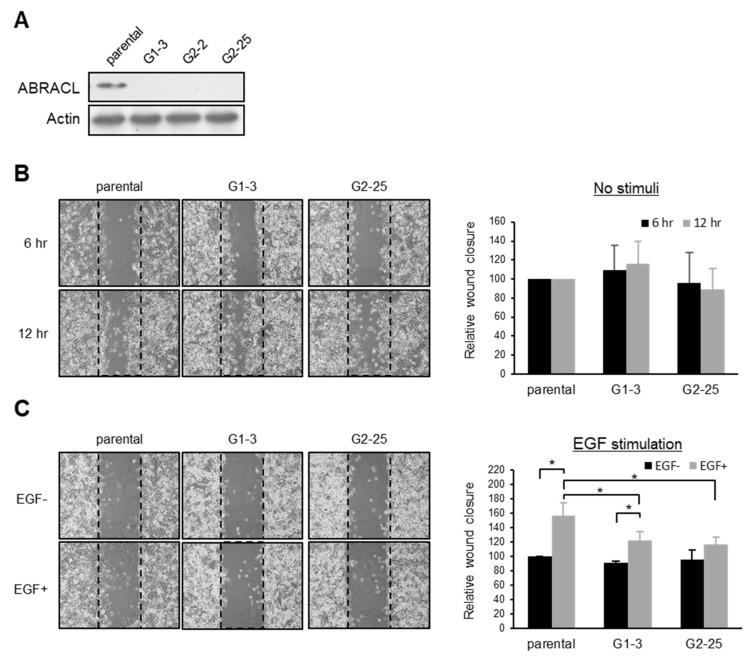
ABRACL modulates elicited cell migration. (**A**) Western analysis on cell lysates from parental and independent CRISPR-mediated *ABRACL*-knockout MDA-MB-231 stable clones (G1-3, G2-2, and G2-25). (**B**–**C**), Wound healing assay. Shown on the left are representative micrographs of the wound regions; parallel black dashed lines indicate the wound edge at 0 h. Quantitative data are shown on the right; the area of the wound region was measured using ImageJ software, and the extent of wound closure relative to that of the parental cells was calculated. Mean ± SD from at least 3 independent experiments are shown; *, *p* < 0.05. (**B**) Cells assayed in a standard medium. (**C**) EGF-stimulated wound healing. Cells were incubated in a serum-free medium for 16 h before an in vitro wound was made at time 0 and further incubated in the absence or presence of 10 ng/mL EGF; images were taken at 0 and 6 h to determine the wound closure efficiency.

The directional migration of cancer cells is stimulated by various signaling molecules, including chemokines and growth factors [3]. For example, it has been reported that EGF promotes the migration of MDA-MB-231 cells [10].To investigate if ABRACL is involved in elicited migratory responses of cancer cells, we employed the EGF-stimulated MDA-MB-231 cell migration assay. CRISPR/Cas9-mediated *ABRACL*-knockout MDA-MB-231 clones were generated (Figure 2A) and subjected to in vitro wound closure cell migration assays. In this cell line, depletion of ABRACL expression did not affect the basal migration activity (Figure 2B). However, when cells were stimulated with EGF in the assay, *ABRACL*-knockout cells exhibited significantly decreased migration compared to the parental control cells (Figure 2C).

Taken together, the above-mentioned results suggest an important role of ABRACL in modulating cancer cell migration.

### 2.2. ABRACL Expression Influences Cell Morphology

**Figure 3:**
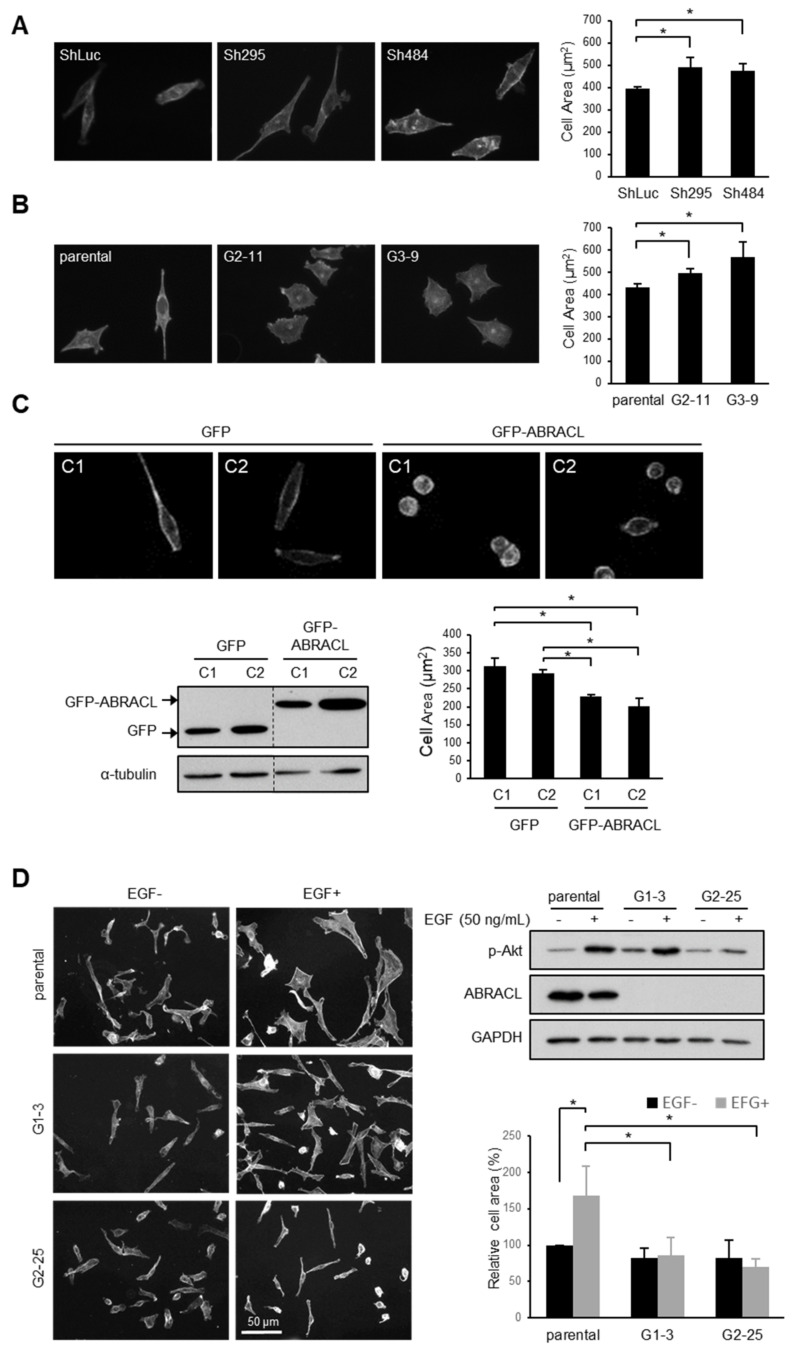
ABRACL expression influences cell morphology. (**A**–**D**) Fluorescence staining and morphology analysis on cells expressing different levels of ABRACL. Cells were fixed and stained for F-actin using TRITC-phalloidin; representative fluorescence micrographs are shown. Microscopic images were captured and subjected to computer-assisted image analysis to calculate the average cell area. Mean ± SD from at least 3 independent experiments are shown; *, *p* < 0.05. (**A**) Control (ShLuc) or *ABRACL*-knockdown (Sh295 and Sh484) stable clones of HCT116 cells. (**B**) Parental or *ABRACL*-knockout (G2-11 and G3-9) HCT116 cells. (**C**) GFP- or GFP-ABRACL-expressing SW620 clones. Western analysis on these clones is also shown. (**D**) Parental and *ABRACL*-knockout (G1-3, G2-25) MDA-MB-231 cells were serum-starved for 16 h and then treated with 50 ng/mL EGF or not for 30 min before analysis. Western analysis on these cells is also shown; the increased p-Akt level demonstrates the effect of EGF.

We noticed that cells expressing different levels of ABRACL displayed different morphology and therefore performed computer-assisted morphology analysis to demonstrate the phenotype. Microscopic images of TRITC-phalloidin-stained cells were captured under a fluorescence microscope and analyzed to determine the cell area. Results showed that compared to the control cells, *ABRACL*-knockdown and *ABRACL*-knockout HCT116 cells were more well-spread on the substratum, exhibiting a larger average cell area (Figure 3A,B). We also analyzed the morphology of GFP- and GFP-ABRACL-expressing clones of SW620 cells (which express low levels of endogenous ABRACL) and found that GFP-ABRACL-expressing clones displayed a significantly smaller average cell area compared to those of the GFP-expressing control clones (Figure 3C).

It is known that EGF can stimulate the spreading of MDA-MB-231 cells [11]. We investigated if ABRACL expression affects cell morphology in EGF-stimulated MDA-MB-231 cells (Figure 3D). In this cell line, there was no obvious effect of ABRACL depletion on the morphology of untreated cells; the average cell areas of *ABRACL*-knockout cells and the parental control cells were similar. We treated cells with EGF and checked the effectiveness of treatment using Akt phosphorylation (p-Akt) as a read-out of the activation of the EGFR-PI3K-Akt signaling pathway, which underlies EGF-induced migration of MDA-MB-231 cells [12]. Results confirmed the pathway-activating effect of EGF in both parental and *ABRACL*-knockout cells. However, while EGF treatment resulted in parental cells displaying a well-spread morphology on the substratum and an increased average cell area, EGF treatment did not induce cell spreading in *ABRACL*-knockout clones, and the average cell area stayed similar to that of untreated cells. These results indicated that ABRACL expression affected the EGF-stimulated morphological change.

Although the effects of perturbing ABRACL expression on cell morphology varied in different cell lines, results together demonstrate that ABRACL expression can affect cell morphology.

### 2.3. ABRACL Associates with the Actin Cytoskeleton

**Figure 4:**
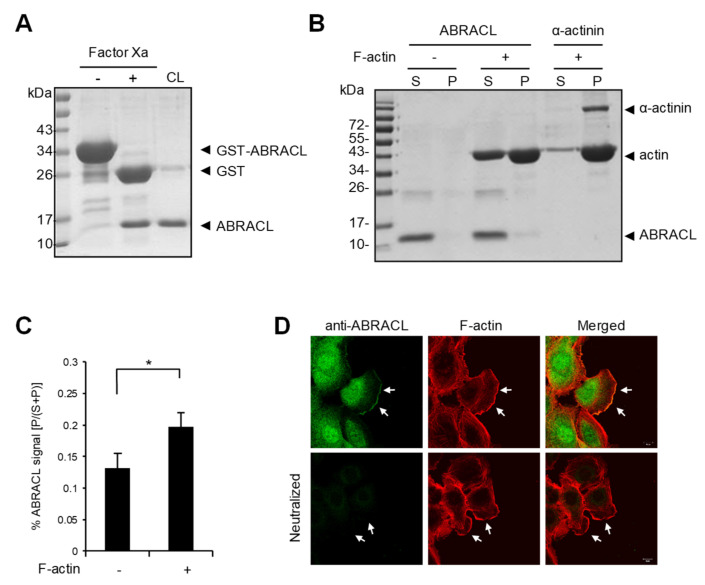
ABRACL associates with the actin cytoskeleton. (**A**) Preparation of recombinant ABRACL. GST-ABRACL purified from *E. coli* was treated with Factor Xa and subjected to clean-up steps to obtain ABRACL. Shown is a gel image after Coomassie blue staining. CL, cleaned-up sample. (**B**) In vitro F-actin sedimentation assay. Non-muscle actin (Cytoskeleton, Inc., Denver, CO, USA) was polymerized into F-actin and incubated with purified ABRACL or α-actinin (Cytoskeleton, Inc., Denver, CO, USA) as a control. After centrifugation to sediment F-actin, supernatant (S) and pellet (P) fractions were analyzed by SDS–PAGE and Coomassie Blue staining. (**C**) Quantitative results of ABRACL co-sedimentation with F-actin. Quantitation of ABRACL signal was done by MultiGauge software. The P/(S+P) signal ratios were calculated. Shown are Mean ± SD from independent experiments shown in (**B**) and Figure S2. (**D**) Colocalization of ABRACL and F-actin. Monolayers of MDA-MB-231 cells were fixed after a scratch “wound” was made and subjected to ABRACL immunofluorescence staining and F-actin staining (using TRITC-phalloidin). In the neutralized control sample, purified ABRACL was added during immunofluorescence staining to neutralize the antibodies. Shown are confocal micrographs. Arrows, lamellipodia.

As the actin cytoskeleton is a major determinant of cell morphology and motility, the aforementioned results are consistent with the notion that ABRACL serves a regulatory function on the actin cytoskeleton in cancer cells as Costars does in *Dictyostelium*. To explore the role of ABRACL in actin regulation, we investigated whether ABRACL interacts with the actin cytoskeleton. We first examined if recombinant ABRACL directly binds to F-actin in vitro. GST-ABRACL purified from *E. coli* was processed by Factor Xa protease digestion, and the cleaved-off GST was removed by GST pulldown procedures to obtain the untagged ABRACL protein (Figure 4A). Using the purified ABRACL in the in vitro F-actin co-sedimentation assay, we found that a small fraction of ABRACL co-sedimented with F-actin (Figure 4B and Figure S2); quantitative results from independent experiments showed a significant increase of ABRACL signal in the F-actin-containing pellet fraction compared to that in the pellet fraction with no added F-actin (Figure 4C). These results indicated that purified recombinant ABRACL bound to F-actin, although very weakly, under the in vitro experimental conditions. We next performed cell staining experiments to examine the cellular localization of ABRACL in relation to that of actin. In MDA-MB-231 cells fixed during the process of in vitro wound closure, fluorescence signals for endogenous ABRACL and F-actin were colocalized at the leading edge of lamellipodia (Figure 4D), suggesting that ABRACL may physically associate with the actin cytoskeleton.

### 2.4. ABRACL Expression Affects the Equilibrium of Cellular F- and G-actin

**Figure 5:**
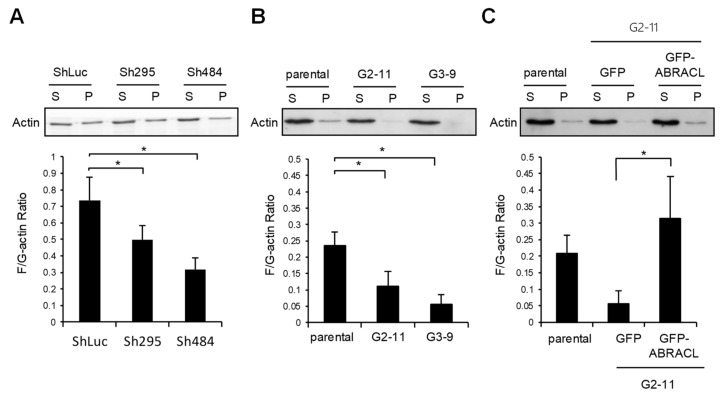
ABRACL expression affects the equilibrium of cellular F- and G-actin. Cell lysates were subjected to G/F-actin fractionation, and amounts of actin in collected supernatant (S; G-actin) and pellet (P; F-actin) fractions were examined by Western analysis and quantitated using MultiGauge software. Shown are results from at least three independent experiments; *, *p* < 0.05. (**A**) Lysates from the control (ShLuc) or *ABRACL*-knockdown (Sh295 and Sh484) HCT116 were analyzed. (**B**) Lysates from parental or *ABRACL*-knockout (G2-11 and G3-9) HCT116 clones were analyzed. (**C**) Lysates from parental or *ABRACL*-knockout HCT116 clone G2-11 cells transfected to express GFP or GFP-ABRACL were analyzed.

We next examined if ABRACL exerts any effect on the steady-state F-actin and G-actin equilibrium. Lysates from control and *ABRACL*-knockdown or *ABRACL*-knockout HCT116 cells were fractionated to obtain F-actin (pellet), and G-actin (supernatant) fractions and the amounts of actin present in each fraction were analyzed. The results showed that both *ABRACL*-knockdown and *ABRACL*-knockout cells displayed lower cellular F-/G-actin ratios compared to the control cells (Figure 5A,B). Moreover, when *ABRACL*-knockout cells were transfected to express GFP-ABRACL, the distribution of cellular actin to the F-actin fraction was increased, resulting in an increased cellular F/G-actin ratio (Figure 5C). These data suggest that ABRACL may modulate actin dynamics in favor of increasing the relative content of F-actin.

### 2.5. ABRACL Interacts with Cofilin

**Figure 6:**
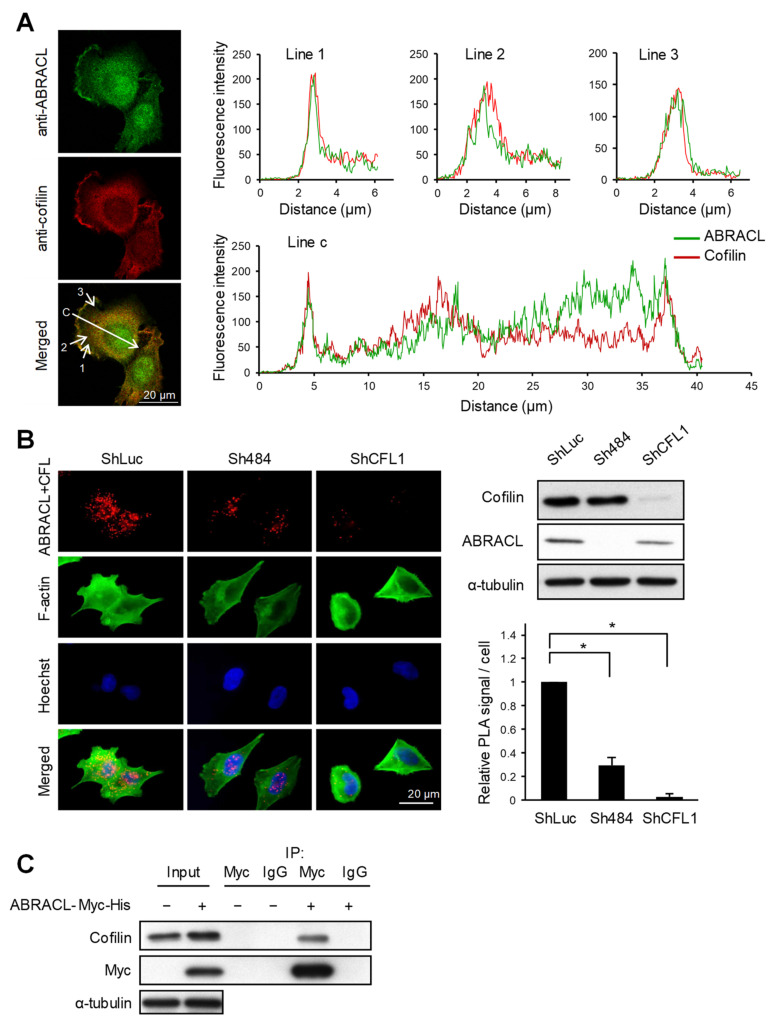
ABRACL interacts with cofilin. (**A**) Immunofluorescence staining. MDA-MB-231 cells were fixed and incubated with both anti-ABRACL and anti-cofilin primary antibodies in the procedure. Fluorescence signals were captured under a confocal microscope. Colocalization of ABRACL and cofilin signals along the shown lines (1, 2, 3, and c) were quantified and plotted against the distance from the origin of each line. (**B**) In situ proximity ligation assay (PLA). HCT116 cells infected with luciferase-targeting (shLuc), *ABRACL*-targeting (Sh484) or cofilin-targeting (shCFL1-1) shRNA-expressing lentivirus were analyzed. Cells were fixed, incubated with anti-ABRACL and anti-cofilin antibodies, and subjected to in situ PLA using the Duolink kit (Sigma-Aldrich). Cells were also stained with FITC-phalloidin for F-actin and Hoechst for DNA. Representative fluorescence micrographs and Western analysis confirming the knockdown effects are shown. Fluorescence signals from PLA were quantitated by counting the signal spots in each cell. Signal intensities relative to the shLuc control sample are shown. *, *p* < 0.01. (**C**) Co-immunoprecipitation assay. Lysates prepared from cells transfected with the control vector (−) or pcDNA3.1Myc/His-ABRACL (+) were subjected to immunoprecipitation using anti-Myc antibodies or IgG (as a negative control). Immunoprecipitated proteins were subjected to SDS–PAGE and Western analysis.

We next explored how ABRACL may participate in regulating actin dynamics. Previously, we identified the well-known actin-depolymerizing factor cofilin [13,14] as a candidate Costars-interacting protein in *Dictyostelium* using co-immunoprecipitation and mass spectrometry analysis (H.-C. Liao and M.-Y. Chen, unpublished results). Given such, we investigated if ABRACL likewise interacts with cofilin and thereby regulates cellular actin dynamics. We first examined if ABRACL and cofilin can colocalize in cells. When randomly migrating MDA-MB-231 cells were fixed and subjected to immunofluorescence staining with ABRACL- and cofilin-specific antibodies, fluorescence images of cells captured by confocal microscopy demonstrated colocalization of ABRACL and cofilin signals; moreover, computer-assisted image analysis showed that the colocalization was particularly evident at lamellipodia (Figure 6A). We further examined the physical interaction of ABRACL and cofilin in situ, employing a proximity ligation assay (PLA) on cells plated on fibronectin. Fluorescence PLA signals were detected in the control cells but greatly diminished in *ABRACL*- or *cofilin*-knockdown cells (Figure 6B), suggesting a specific interaction of ABRACL and cofilin in cells. When immunoprecipitation was performed in lysates from cells expressing ABRACL-Myc-His using anti-Myc antibodies, endogenous cofilin was detected together with the immunoprecipitated ABRACL-Myc-His (Figure 6C), confirming that ABRACL can associate with cofilin in cells.

### 2.6. ABRACL Inhibits Cofilin-Mediated Actin Depolymerization In Vitro

**Figure 7:**
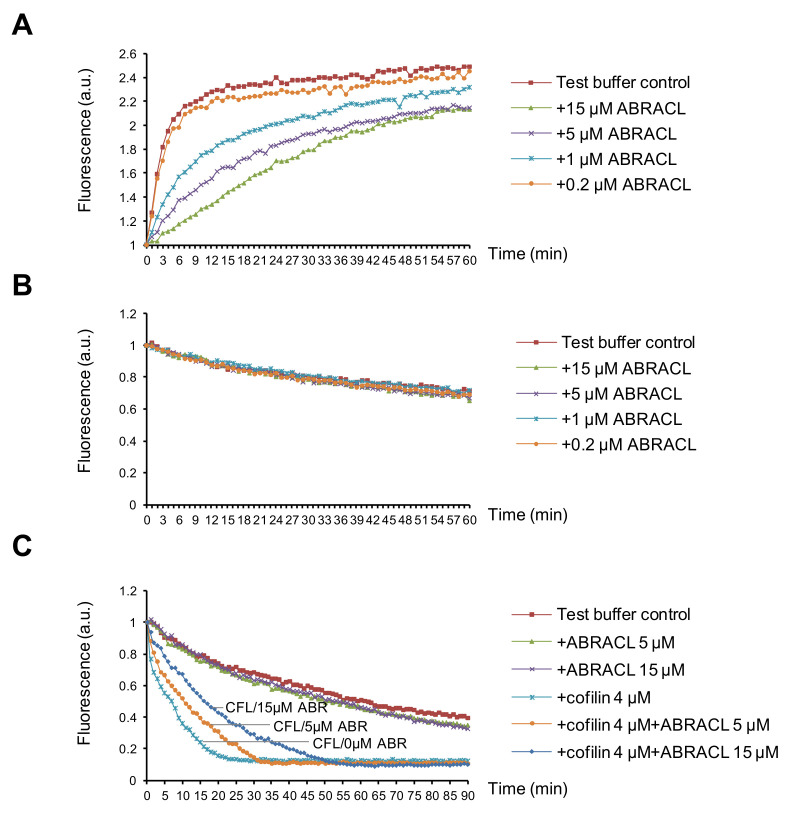
ABRACL can affect actin dynamics in vitro. (**A**) In vitro actin polymerization assay. Pyrene G-actin was incubated with test buffer alone or with indicated concentrations of purified recombinant ABRACL. The intensity of emitted fluorescence in each mixture was monitored using the TECAN-infinite reader. Shown are normalized fluorescence intensities in arbitrary units (a.u.) in relation to the incubation time after initiating polymerization by 1X actin polymerization buffer at time 0. (**B**–**C**), In vitro actin depolymerization assay. Preformed pyrene F-actin was incubated with purified ABRACL alone at indicated concentrations (**B**) or with cofilin (CFL) in the absence or presence of ABRACL (ABR) at indicated concentrations (**C**), and the intensity of emitted fluorescence was monitored. Shown are normalized fluorescence in relation to the incubation time after adding test proteins at time 0.

To further examine the effects of ABRACL on actin dynamics, we tested purified recombinant proteins in in vitro actin assays. When only purified recombinant ABRACL was added in the actin polymerization reactions, the results showed that ABRACL inhibited actin polymerization in a dose-dependent manner (Figure 7A). When tested in the depolymerization reactions, ABRACL alone did not affect the kinetics of actin depolymerization even at a concentration as high as 15 μM in the reaction (Figure 7B). These in vitro results appeared to contradict the above-mentioned finding of decreased F/G-actin ratios in cells lacking ABRACL; one possible explanation for this is that in the cellular context, ABRACL may be exerting its actions together with its interacting proteins. Given the physical interaction between ABRACL and cofilin, we investigated their functional interaction. Cofilin is an actin-binding protein, which multiplies the number of depolymerizing actin ends by severing activity and enhances the pointed end depolymerization and thereby increases actin filament disassembly [14,15]. We employed the in vitro actin depolymerization assay to examine if ABRACL affects the activity of cofilin. Results showed that adding cofilin into preformed pyrene-F-actin accelerated the decay of the fluorescence signal, and the addition of ABRACL in the reaction blunted this effect of cofilin in a dose-dependent manner (Figure 7C). Because binding of cofilin to F-actin can cause quenching of the fluorescence of pyrene F-actin [16,17], to rule-out that the above finding was caused by ABRACL inhibiting the binding of cofilin to F-actin, we performed the in vitro F-actin co-sedimentation assay to test the possibility. We found that ABRACL did not inhibit the co-sedimentation of cofilin with F-actin (Figure S3). Taken together, these findings suggest that ABRACL can functionally interact with cofilin in modulating actin dynamics, consistent with the scenario in which ABRACL promotes the distribution of cellular actin to the F-actin fraction by inhibiting cofilin-stimulated actin disassembly.

### 2.7. ABRACL Is Overexpressed in Human Cancerous Tissues, and the Depletion of ABRACL Expression Reduces Cell Proliferation and Tumorigenic Growth

**Figure 8:**
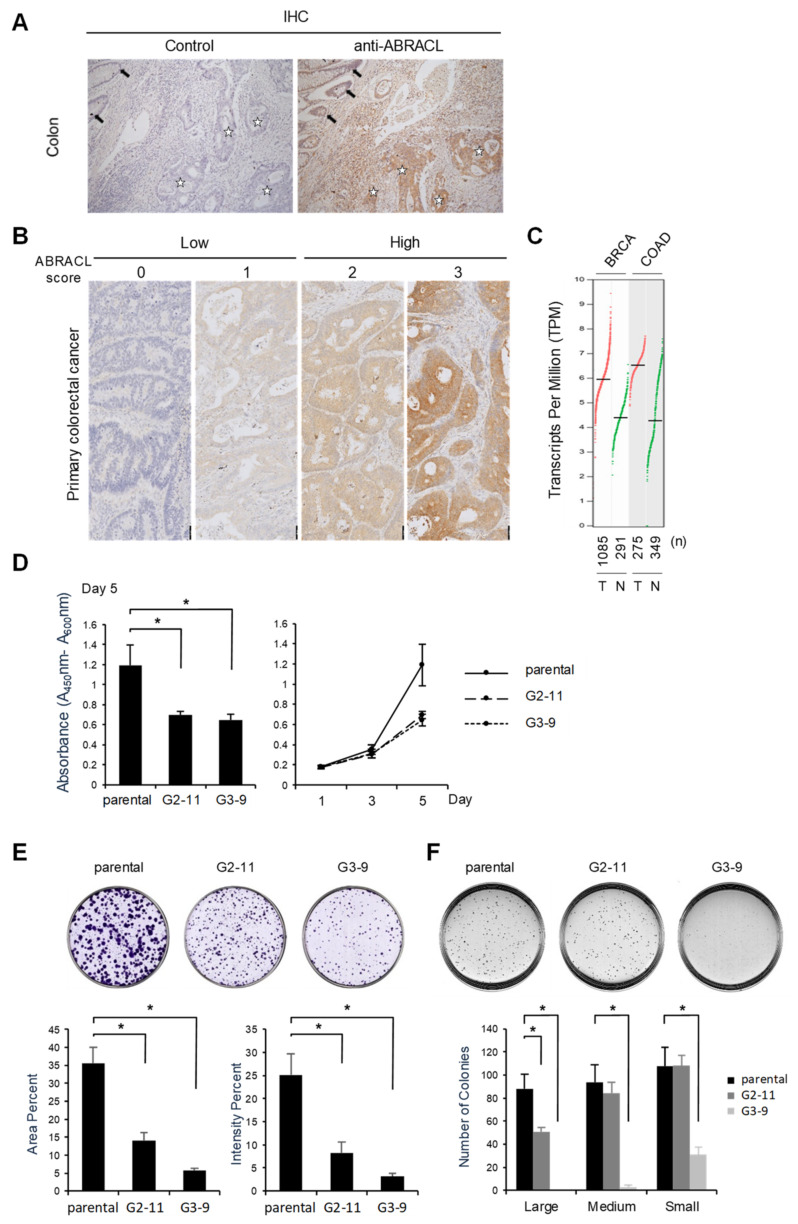
ABRACL is overexpressed in cancerous tissues, and depleting its expression reduces cell proliferation and tumorigenic growth. (**A**–**B**) Immunohistochemistry (IHC) analysis. (**A**) Tissue sections of colorectal carcinoma were analyzed by IHC without (control) or with anti-ABRACL primary antibodies. Stars, representative tumor tissue; arrows, adjacent non-tumor tissue. (**B**) Representative images showing the scoring of IHC results. Scores of 0–1 and 2–3 were defined as low and high expression of ABRACL, respectively. (**C**) *ABRACL* transcript levels in the corresponding tumor (T) and normal (N) tissues. The chart was derived using the GEPIA server, analyzing data from the Cancer Genome Atlas (TCGA) and Genotype-Tissue Expression (GTEx) databases. Cancer types are indicated on the top: BRCA, breast invasive carcinoma, COAD, colon adenocarcinoma. Red and green dots represent data from cancerous and normal tissues, respectively; total numbers (*n*) of samples in each set are shown below the chart. Statistical analyses of the results indicated that *ABRACL* expression was significantly different (*p* < 0.05) between cancerous and normal samples in both cancer types. (**D**–**F**), Parental and stable *ABRACL*-knockout clones (G2-11, G3-9) of HCT116 cells were analyzed. Quantitative results were from at least 3 independent experiments. (**D**) Colorimetric cell proliferation assay. The viability of cells was examined using the WST-1 assay. Shown are relative absorbance values at day 5 (left) and plots of the time course of cell growth (right). *, *p* < 0.05. (**E**) Colony formation assay. Colonies were analyzed on day 10. (**F**) Anchorage-independent growth assay. Colonies were analyzed on day 15. Shown are quantitative results of colonies in different ranges of the area: small, 0.01 to 0.05 mm^2^; medium, more than 0.05 and less than 0.1 mm^2^; large, more than 0.1 mm^2^. *, *p* < 0.05.

As cell migration is deemed pivotal in cancer pathogenesis, and because our findings suggested that ABRACL may regulate cell motility through modulating actin dynamics, we were prompted to investigate the expression of ABRACL in clinical specimens. Immunohistochemistry (IHC) analysis on human colon cancer tissue sections showed strong ABRACL immunoreactivity in the tumor but not in adjacent non-tumor tissues (Figure 8A). We examined more specimens in tissue microarrays of primary colon cancers for ABRACL expression using IHC, and the staining was scored by pathologists (Figure 8B); when the data were analyzed in relation to clinical records of patients, results revealed a significantly higher rate of high ABRACL expression in cases with distant metastasis (41.4%) than that in cases with no metastasis (11.1%) (*p* < 0.01) (Table 1). Such findings were consistent with the notion that ABRACL may contribute to cancer metastasis by enhancing cell motility. Given the high ABRACL protein level in cancer, we asked whether *ABRACL* is upregulated at the transcript level. Bioinformatics surveys using the available data of RNA sequencing expression profiles on clinical samples from the TCGA and GTEx projects in the GEPIA web server (http://gepia.cancer-pku.cn.html, September 2020) revealed that *ABRACL* transcript levels were upregulated, with statistical significance, in various types of cancers compared to respective normal tissues (Figure 8C and Figure S4).

Having noted the differential ABRACL expression in normal and cancerous tissues, we next investigated whether ABRACL expression may also be associated with tumorigenesis. Stable *ABRACL*-knockout HCT116 clones were subjected to WST-1 assays, and the results demonstrated that cell proliferation was inhibited in *ABRACL*-knockout cells when compared to the control (Figure 8D). The depletion of ABRACL expression in HCT116 cells also caused a decrease in tumorigenic growth and the extent of transformation, as evidenced by results of the in vitro colony formation assays (Figure 8E) and anchorage-independent growth assays on the soft agar plates (Figure 8F). Taken together, these data suggest that ABRACL plays an important role in maintaining the tumorigenic potential of cancer cells.

## 3. Discussion

Our characterization of the cellular functions of ABRACL in this study has demonstrated that ABRACL is a conserved regulator of actin and cell migration. The low-migration phenotype of *Dictyostelium* cells with a knockout allele of the gene encoding the ABRACL ortholog Costars [6] was recapitulated in human cancer cells depleted of ABRACL expression. Our data also suggest that ABRACL, just like *Dictyostelium* Costars, can associate with the actin cytoskeleton, localize to the leading edge of migrating cells, and interact with cofilin. Together, these observations support the possibility that ABRACL and its orthologs serve a fundamental molecular function in regulating cellular actin dynamics and thereby modulating cell motility.

Although ABRACL resembles the F-actin-binding domain of ABRA, the binding of purified recombinant ABRACL to F-actin observed in in vitro co-sedimentation assays was not robust. It has been reported that two separate yet co-dependent regions (aa 234–279 and 346–375) in ABRA are required for its actin-binding; deleting either region abolishes the association of ABRA with F-actin [7]. Structural and functional dissection of ABRA has further defined two F-actin-binding domains, ABD1 (aa 193–296) and ABD2 (aa 294–375); both domains can bind F-actin independently when tested in vitro, but ABD2 binds with a much lower affinity than ABD1 [18]. The protein sequence of ABRACL exhibits homology mostly to ABD2, with ~40% identities and ~60% similarities to aa 326–381 of ABRA in the Blast-2 protein analysis (https://blast.ncbi.nlm.nih.gov (accessed on 31 January 2021)), which may explain the weak in vitro binding of ABRACL to F-actin. We have noted that Lin et al. did not detect the association between actin and ABRACL [9]; possible explanations for this discrepancy include that the amounts of test proteins used in the assay were too small to see the small fraction of ABRACL in the co-sedimentation sample or that the His-tag in the recombinant ABRACL used had interfered with the interaction with F-actin.

Given the weak direct interaction of ABRACL with F-actin, we suppose that the localization of ABRACL to the F-actin-rich lamellipodia front may depend on its interaction with other actin regulators. It has been reported that the actin-binding domain of ABRA interacts with another two F-actin-binding proteins, i.e., ABLIM2 and ABLIM3 [19]. However, despite the homology of ABRACL to the ABD2 domain of ABRA, we did not detect any interaction of ABRACL with these ABLIM proteins (W.-T. Kao and M.-Y. Chen, unpublished). Instead, we have found that ABRACL can interact with cofilin, and they colocalize nicely at the lamellipodia in this study. As Cofilin is known to bind to F-actin [13], it is conceivable that ABRACL localizes to the F-actin-rich region through interacting with cofilin. Nevertheless, as a comprehensive search for ABRACL-interacting proteins was not performed in this study, we cannot exclude the possibility that there exist other F-actin-binding or lamellipodia-localizing cellular proteins responsible for the localization of ABRACL to the leading edge of migrating cells.

The effects of Costars family members on F- and G-actin homeostasis appear to be dependent on the cellular context. Our previous work has demonstrated that the depletion of the *Dictyostelium* Costars increases the cellular F-actin content [6], while in this study, we found that the suppression of ABRACL expression lowered the F/G-actin ratio in cancer cells. These opposing observations are unlikely caused by a difference in the intrinsic activity of the two orthologs. This is because the expression of the human ABRACL can rescue phenotypes of *Dictyostelium* cells lacking Costars [6], which indicates that human ABRACL and Costars function similarly in the context of *Dictyostelium* cells. A probable explanation for the different effects between depleting ABRACL and Costars on actin dynamics is that the repertoires of actin regulators in these cells are different. Findings in this study do suggest that ABRACL may coordinate with other cellular actin regulators, of which a good candidate is cofilin, to cause a net effect of promoting the distribution of cellular actin to the polymerized pool. While the expression of ABRACL in *ABRACL*-knockout cells elevated the F-actin levels, the purified ABRACL protein alone did not promote actin polymerization or inhibit F-actin depolymerization in vitro; instead, the presence of ABRACL decelerated the cofilin-stimulated F-actin depolymerization in vitro.

Given the evidence of physical interaction between ABRACL and cofilin we obtained in this study, it is also possible that ABRACL exerts its cellular function by modulating the activity of cofilin. Cofilin has multifaceted functions on actin dynamics; it can sever the actin filaments and promote F-actin depolymerization, while, under certain circumstances, also facilitate actin polymerization via generating free barbed ends and increasing the pool of actin monomers [13,14,20,21,22]. As the actin cytoskeleton is temporally and spatially dynamic in living cells, it is not surprising that cofilin is tightly controlled by signaling pathways and regulatory molecules in achieving its specific spatiotemporal activation. For example, the phosphorylation status of the Ser-3 residue of cofilin, which is regulated by specific kinases and phosphatases, such as LIMK1 and Slingshot, can control the activity of cofilin [23,24]. Cofilin is also negatively regulated via binding to membrane phosphatidylinositol 4,5-bisphosphate (PIP_2_), and hence, signaling events-induced local hydrolysis of PIP_2_ can result in spatially-confined activation of cofilin [25,26]. Studies have also demonstrated the modulation of cofilin activity by other actin-binding proteins; examples include the cyclase-associated proteins CAP1 and CAP2, which bind to F-actin and enhance cofilin-mediated actin severing [27], and coactosin-like 1 (COTL1), which also associates with F-actin, but antagonizes cofilin-mediated actin depolymerization [28]. Here we have demonstrated in vitro that ABRACL can pose a negative effect on the activity of cofilin. Determining whether ABRACL represents another cellular regulator of cofilin requires further investigation.

This study has uncovered a role of ABRACL in supporting optimal cell proliferation, which was not previously discovered for *Dictyostelium* Costars, and has also established an association of ABRACL expression with tumorigenic growth. To date, very few reports have mentioned high expression of ABRACL in cancers. A proteomic approach for biomarkers previously found ABRACL in the uterine aspirates of patients with endometrial cancer, but not in aspirates from healthy subjects [29]. Another immunohistochemistry study discovered that ABRACL was expressed at higher levels in cancerous than in normal gastric tissues, and high ABRACL expression was associated with poor clinical outcomes [30]. However, our bioinformatics analysis suggests the upregulation of *ABRACL* as a common molecular alteration associated with cancer pathogenesis, as it is observed in various types of cancer. The functional significance of ABRACL in tumorigenesis and cancer progression is supported by our findings that ABRACL is overexpressed in cancerous tissues and that cancer cells with ABRACL expression, rather than cells depleted of ABRACL, migrate more robustly, proliferate better, and are more capable of generating colonies even under anchorage-independent conditions.

The molecular mechanisms involved in the function of ABRACL in tumorigenesis remain to be elucidated. An important issue to address in future investigations is whether such mechanisms are dependent on the activity of ABRACL in regulating the actin dynamics. There is increasing evidence in the literature to support that actin and actin regulators play important roles in the mechanisms underlying carcinogenesis and cancer progression. In addition to the involvement of dynamic actin remodeling in forming cellular protrusions to support the migration and invasion of cancer cells during cancer progression, it has also been established that actin has essential functions in fundamental nuclear processes, such as transcription, chromatin remodeling, RNA processing, DNA replication, and DNA repair [31,32,33,34]; dysregulation of these processes often occurs in cancer pathogenesis. Of those which have been found in the nucleus, dozens of actin-binding proteins serve to regulate actin dynamics and control the levels of nuclear actin monomers and polymers [31]; interestingly, cofilin is among these nuclear actin regulators. Other than being involved in the regulation of actin dynamics, cofilin can interact with importin-9 and facilitate the transport of actin into the nucleus [35], and also play a role in the RNA polymerase II transcriptional machinery [36]; perturbation of these cofilin functions may affect the cellular transcriptional program and lead to pathogenic changes. The role of ABRACL in regulating functions of nuclear cofilin and nuclear actin warrants further exploration.

In conclusion, this study demonstrates that the human Costars family protein ABRACL has the conserved function in regulating actin and cell migration and also highlights ABRACL as a molecular player in cancer pathogenesis.

## 4. Materials and Methods

### 4.1. Cell Culture and Transfection

Cell lines were originally obtained from Bioresource Collection and Research Center, Taiwan. Cells were cultured in media containing 10% fetal bovine serum (FBS) at 37 °C with 5% CO_2_. MDA-MB-231 cells were cultured in DMEM-F12 (Gibco, Grand Island, NY, USA) with the supplement of non-essential amino acids and L-glutamine (2 mM). HCT116 cells were cultured in the McCoy’s 5A medium (Sigma-Aldrich, St. Louis, MO, USA), while SW620 cells were cultured in the Leibovitz’s L-15 medium (Gibco, Grand Island, NY, USA). For transfection, lipofectamine 2000 was used according to the manufacturer’s instruction (Invitrogen, Carlsbad, CA, USA).

### 4.2. Plasmids and Primers

Plasmids and primers used in this study are listed in Table S1.

### 4.3. Lentivirus-Delivered shRNA-Mediated Gene Knockdown

The expression of *ABRACL* or the cofilin gene (*CFL1*) was silenced using a lentivirus-based method following the protocol suggested by the RNA Technology Platform and Gene Manipulation Core (Academia Sinica, Taipei, Taiwan). Two *ABRACL*-targeting short hairpin sequences (Sh295 and Sh484) and one *CFL1*-targeting short hairpin sequence (shCFL1-1) were cloned into pLKO plasmid. Each shRNA-expressing plasmid was mixed with two other vectors, pMD.G, which expresses VSV-G envelope glycoprotein and pCMV∆R8.91, which contains *gag*, *pol* and *rev* genes for packaging lentivirus, and transfected into 70% confluent 293 T cells for lentivirus production. After 18 h, the culture medium was replaced with 1% BSA (Sigma-Aldrich, St. Louis, MO, USA)-containing DMEM (Gibco, Grand Island, NY, USA) to improve virus yield. Culture medium containing lentivirus was collected and tested for relative infection unit by cell viability assay. For gene knockdown, cells were infected with the shRNA-expressing lentivirus and selected in puromycin (1 μg/mL) for 3 days and subsequently examined for ABRACL or cofilin expression by Western analysis.

### 4.4. CRISPR/Cas9-Mediated ABRACL Knockout

The knockout of the *ABRACL* gene was performed using a CRISPR/Cas9-based method previously described [34]. An *ABRACL*-targeting gRNA-expressing vector and a Cas9-expressing vector were co-transfected into HCT116 or MDA-MB-231 cells using lipofectamine 2000 (Invitrogen, Carlsbad, CA, USA). Two *ABRACL*-targeting gRNA sequences were used: G2 (5′-GCGAGGTTAACCTCTTAGTGG-3′) and G3 (5′-GATGAATTTCCTCCACTAAG-3′). Transfected cells were allowed to recover for 48 h and subsequently re-plated at a density of ~50 cells/10 cm dish. After incubation for 15 days, colonies were collected and examined for ABRACL expression by Western analysis. Candidate clones with no detectable ABRACL expression were further confirmed by sequencing the PCR amplification products of the *ABRACL* genomic locus.

### 4.5. Migration Assays

For the Transwell migration assay, 2–5 × 10^5^ HCT116 cells in serum-free medium or 5 × 10^4^ MDA-MB231 cells in 0.5% FBS-containing medium were seeded in the upper chamber of Millicell insert (Merck KGaA, Darmstadt, Germany) and incubated for 6 or 12 h with 10% FBS-supplemented medium in the lower chamber. Cells that migrated across the membrane in the insert were fixed, stained with crystal violet and counted. The migration index was calculated by normalizing the number of migrating cells in each test sample to that obtained in the control sample.

For the in vitro wound healing migration assay, 1 × 10^5^ MDA-MB231 cells were seeded into the culture insert (cat. no. 80209; ibidi, Gräfelfing, Germany) and allowed to adhere. The culture insert was removed to make an in vitro “wound”, and images of the wound area were taken at 0, 6, and 12 h, respectively. To assess the EGF-stimulated cell migration, seeded cells were incubated in serum-free medium for 16 h before the removal of the culture insert and the change to fresh serum-free medium or EGF (10 ng/mL)-supplemented medium at time 0; micrographic images of the wound area were taken at 0 and 6 h, respectively. The area of the wound region was measured using ImageJ software, and the extent of wound closure relative to that of the parental cells was calculated.

### 4.6. Cell Morphology Analysis

Cells were cultured on fibronectin-coated coverslips and fixed in 3.7% paraformaldehyde in 1X phosphate-buffered saline (PBS). Cells were subsequently permeabilized in 0.2% Triton X-100 in PBS and stained in a 50 μg/mL TRITC-phalloidin (Sigma-Aldrich, St. Louis, MO, USA) solution in PBS at room temperature. Cell morphology and actin organization were examined using differential interference contrast (DIC) and fluorescence microscopy. Microscopic images of stained cells were captured and analyzed using either Zeiss AxioVision or ImageJ software to calculate the average cell area. At least 3 independent experiments were performed, and a total of more than 150 cells were analyzed for each cell clone or test condition.

### 4.7. Purification of Recombinant Proteins for In Vitro Assays

Recombinant GST and GST-ABRACL proteins were expressed in *E. coli* and purified by GST pulldown procedures using glutathione sepharose beads (Sigma-Aldrich; St. Louis, MO, USA). To remove the GST tag, each 300 μg sample of GST-ABRACL was incubated with 1 μg of factor Xa at 4 °C for 12 h; the digestion reaction was mixed with the factor Xa removal resin (Qiagen, Frederick, MD, USA) to stop the reaction and remove factor Xa and subjected to another round of GST-pulldown procedures to remove GST. Recombinant His-cofilin was expressed in *E. coli* and purified by His tag pulldown procedures using Ni^2+^-NTA agarose beads (Millipore, Darmstadt, Germany). Preparations of purified proteins were placed in Amicon Ultra- 0.5 mL centrifugal devices to remove the elution buffer and equilibrate in an appropriate assay buffer.

### 4.8. In Vitro F-Actin Co-Sedimentation Assay

The interaction between ABRACL and F-actin was tested in this co-sedimentation assay. Rabbit skeletal muscle actin (Cytoskeleton, Inc., Denver, CO, USA) was diluted to 0.1 mg/mL, incubated on ice for 30 min and subsequently allowed to polymerize in 1X actin polymerization buffer (50 mM KCl, 2 mM MgCl_2_ and 1 mM ATP in 10 mM Tris, pH 7.5) at room temperature for 1 h. Purified recombinant ABRACL was mixed with the F-actin formed in the polymerization reaction and incubated at room temperature for 30 min; a sample of α-actinin purified from rabbit skeletal muscle (Cytoskeleton, Inc., Denver, CO, USA) was also mixed with the F-actin as a positive F-actin-binding control. After centrifugation at 150,000× *g* for 90 min, equivalent fractions of supernatants and pellets were subjected to SDS–PAGE and the gel was stained with Coomassie blue to visualize the separated proteins.

### 4.9. Analysis of the Cellular F/G-Actin Ratio

Cells were lysed in a lysis buffer (50 mM PIPES pH 6.9, 50 mM NaCl, 5 mM MgCl_2_, 5 mM EGTA, 5% (*v*/*v*) glycerol, 0.1% NP-40, 0.1% Triton X-100, 0.1% Tween-20, 0.1% 2-mercaptoethanol) freshly supplemented with 100 mM ATP and 1X protease inhibitor (Roche, Mannheim, Germany) before use. Aliquots of lysates containing equal amounts of cellular proteins were subjected to centrifugation at 2000× rpm for 15 min to eliminate the cell debris, and the supernatant fraction from each sample was subjected to high-speed centrifugation (55,000× rpm in a TLA100.3 rotor of Beckman Coulter Inc.) for 1 h. The resulting supernatant (G-actin) and pellet (F-actin) fractions were examined for the amount of actin using Western analysis. Actin signals in G- and F-actin fractions were quantitated using MultiGauge software to determine the F/G-actin ratio. Relative F/G-actin ratios were calculated by normalizing to the ratio of the control sample.

### 4.10. Immunofluorescence Cell Staining

Cells were cultured on fibronectin-coated coverslips, fixed in 3.7% paraformaldehyde in PBS, and permeabilized in 0.2% Triton X-100 in PBS. After incubation in the blocking solution (10% FBS in PBS) for 1 h, cells on the coverslip were incubated with rabbit anti-ABRACL (1:400; Sigma-Aldrich Cat. #HPA030217, St. Louis, MO, USA) and mouse anti-cofilin primary antibodies (1:400; Santa Cruz E-8, Dallas, TX, USA), and subsequently with FITC-conjugated anti-rabbit IgG and TRITC-conjugated anti-mouse IgG secondary antibodies, respectively. Fluorescence signals were captured under a Zeiss LSM 700 confocal microscope. Colocalization of fluorescence signals was analyzed using MetaMorph software.

### 4.11. Duolink In Situ Proximity Ligation Assay (PLA)

Duolink™ PLA (Sigma-Aldrich, St. Louis, MO, USA) was used for detecting the interaction between endogenous ABRACL and cofilin in situ within the cell. In this assay, two test proteins are first recognized by individual primary antibodies raised in different species. Next, two secondary antibodies conjugated with specific DNA-oligonucleotide (i.e., PLA probes) are added to bind to individual primary antibodies. If the two proteins of interest are close enough in situ, a connector oligonucleotide added into the reaction will hybridize and join the PLA probes to form a circular DNA structure. Following a ligation step, the circular DNA template can be amplified by DNA polymerase via a rolling-circle amplification process to generate copies of DNA for subsequent detection by fluorochrome-coupled probes [37]. For observing the interaction between ABRACL and cofilin in situ, cells were cultured on fibronectin-coated 18 mm chamber slides for 24 h before being fixed in 3.7% paraformaldehyde in PBS and permeabilized in 0.2% Triton X-100 in PBS. After incubation in the blocking solution for 1 h at 37 °C, washed in PBS, cells on the slides were incubated overnight with rabbit anti-ABRACL (1:400; Sigma-Aldrich Cat. #HPA030217, St. Louis, MO, USA) and mouse anti-cofilin primary antibodies (1:400; Santa Cruz E-8, Dallas, TX, USA) at 4 °C. After washing in TBST, PLA probes were added to the slides and PLA was performed following the manufacturer’s instructions (Sigma-Aldrich, St. Louis, MO, USA). The Duolink™ fluorescent detection red (Sigma-Aldrich, St. Louis, MO, USA) was used for the visualization of protein interaction. The slides were subsequently subjected to Hoechst staining for DNA or stained with FITC-phalloidin to visualize F-actin.

### 4.12. In Vitro Actin Polymerization and Depolymerization Assays

A commercially available kit (Cytoskeleton, Inc. Cat. no. BK003, Denver, CO, USA) was used to assess the effects of purified recombinant proteins on actin polymerization or depolymerization. For the polymerization assay, pyrene G-actin was freshly prepared following the manufacturer’s protocol and 200 μL aliquots of 0.4 mg/mL pyrene G-actin were loaded onto a 96-well plate placed in the fluorimeter (TECAN-Infinite 200 PRO, Tecan Trading AG, Switzerland). The emitted fluorescence was recorded every min. After monitoring the baseline fluorescence for 3 min, 20 μL samples of purified ABRACL (or test buffer as a control) were added, and the mixtures were further incubated for 25 min. Next, 20 μL of 10X actin polymerization buffer was added to activate actin polymerization, and the reading was continued until 2 h. For the depolymerization assay, pyrene F-actin samples were freshly prepared following the manufacturer’s instruction and diluted to 0.2 mg/mL. Aliquots (200 μL) of pyrene F-actin were mixed with purified recombinant proteins (or test buffer as a control), and the mixtures were loaded onto a 96-well plate. The emitted fluorescence was taken every min by the fluorimeter for 2 h. For plotting the results of both assays, the average value of the recorded fluorescence at three time points before adding purified protein (ABRACL and/or cofilin) was calculated, and the fluorescence readings at the following time points were normalized to this average value to eliminate the basal fluorescence difference between samples.

### 4.13. Clinical Tissue Samples

A total of 147 formalin-fixed, paraffin-embedded clinical specimens of surgically resected primary colorectal carcinomas were obtained without any bias of gender or age from the archived tissues at the Taipei Veterans General Hospital (Taipei-VGH). For constructing the tissue microarray (TMA), representative areas of the tumor were selected, and a 3 mm tissue core was retrieved from the paraffin block of each case. Clinical data were collected retrospectively and received as de-identified patient data from the hospital-based data registry. The study protocol was approved by the Ethics Committee of the Taipei-VGH, Taiwan (IRB No. 2020-04-011BC).

### 4.14. Immunohistochemistry (IHC) Analysis

TMA sections (5 μm) were deparaffinized, de-waxed and dehydrated through xylene and graded alcohol treatments, immersed in the antigen-retrieval buffer, processed for endogenous peroxidase blocking, and incubated with the primary antibody (1:400; rabbit anti-ABRACL; Sigma-Aldrich Cat. #HPA030217, St. Louis, MO, USA). The specimens were then processed through the polymer detection system (Novolink Polymer detection kit; Leica Biosystems, Buffalo Grove, IL, USA), dehydrated and counterstained with hematoxylin. The expression of ABRACL was scored independently by pathologists at the Veterans General Hospital, Taipei. The level of ABRACL was graded by the intensity of staining (0, absent; 1, weak; 2, moderate; and 3, strong) (Figure 8B). The scores of 0–1 and 2–3 were defined as low and high expression, respectively.

### 4.15. Cell Proliferation Assay

The WST-1 assay was performed according to the manufacturer’s instructions (Roche, Mannheim, Germany). The WST-1 reagent is a tetrazolium salt, which can be converted to a colored product formazan by mitochondrial enzymes in living cells. Therefore, the amount of the colored product generated is proportional to the number of viable cells, and spectrophotometric quantification of the product can be used for the measurement of cell proliferation and viability. In this assay, cells were seeded (at 1000 cells/well) in 96-well plates in triplicates and grown for 1–5 days at 37 °C in 5% CO_2_. At each time point, WST-1 reagent was added to each well and mixed by shaking the plate on a rotary shaker at 150× rpm; the mixtures were incubated for 2 h before values of absorbance at 450 nm (test wavelength), and 690 nm (reference wavelength) were measured to determine the percentage of live cells.

### 4.16. Colony Formation Assay

Cells were seeded at a density of 1000 cells/well on 6-well plates and allowed to grow for 10 days. Colonies were stained with 5% Giemsa (Sigma-Aldrich, St. Louis, MO, USA). Areas and staining intensity of the colonies were analyzed using ImageJ software equipped with the ColonyArea plug-in.

### 4.17. Anchorage-Independent Growth Assay

Cells (1000 cells/dish) in 0.4% low melting point agarose were plated on top of a 1% agar layer in 3.5 cm culture dishes and incubated for 15 days. Colonies were fixed in methanol, stained with crystal violet, and analyzed using the ImageJ software.

### 4.18. Statistical Analysis

Statistical analysis of ABRACL expression in clinical specimens was performed using SPSS 17.0 software. Continuous and categorical variables were evaluated by Student’s *t*-test and Pearson’s chi-squared (χ^2^) test, respectively. All reported *p*-values were for two-sided tests, and results with a *p*-value smaller than 0.05 were considered statistically significant.

## Figures and Tables

**Table 1:** ABRACL expression in 147 primary colorectal cancer specimens.

Clinicopathological Characteristics		ABRACL Expression ^a^				*p* Value ^c^
		High		Low		
Group	*n*	*n*	(%)	*n*	(%)	
Total	147					
Gender						
Male	86	29	(33.7)	57	(66.3)	NS ^d^
Female	61	21	(34.4)	40	(65.6)	
Age ^b^ (y)						
66 and older	68	24	(35.3)	44	(64.7)	NS ^d^
Less than 66	79	26	(32.9)	53	(67.1)	
Metastasis						
M(+)	111	46	(41.4)	65	(58.6)	0.002 **
M(−)	36	4	(11.1)	32	(88.9)	

^a^ Intensity of staining was scored by pathologists (0, absent; 1, weak; 2, moderate; and 3, strong; representative examples are shown in Figure 8B); the scores of 0–1 and 2–3 were defined as low and high expression, respectively. ^b^ Divided into 2 groups by the median age; ^c^ χ^2^ test; ^d^ not significant (*p* > 0.05); ** statistically significant (*p* < 0.01).

## Data Availability

The data presented in this study are available on request from the corresponding author.

## Supplementary Materials

The following are available online at https://www.mdpi.com/1422-0067/22/4/2037/s1. Table S1. Plasmids and primers used in this study. Figure S1. ABRACL expression in human cancer cell lines; Figure S2. In vitro F-actin co-sedimentation assay; Figure S3. Testing ABRACL and cofilin in in vitro F-actin co-sedimentation; Figure S4. Upregulation of the *ABRACL* transcript level in various types of cancer.

## Abbreviations

ABRA	Actin-binding rho-activating protein
ABRACL	ABRA *C*-terminal like
BSA	Bovine serum albumin
CFL	Cofilin
CRISPR	Clustered regularly interspaced short palindromic repeats
EGF	Epidermal growth factor
EGTA	Ethylene glycol tetraacetic acid
FBS	Fetal bovine serum
FITC	Fluorescein isothiocyanate
GST	Glutathione *S*-transferase
GFP	Green fluorescent protein
IHC	Immunohistochemistry
PBS	Phosphate-buffered saline
PCR	Polymerase chain reaction
PIPES	Piperazine-*N*,*N*′-*bis*(2-ethanesulfonic acid)
PLA	Proximity ligation assay
SDS–PAGE	Sodium dodecyl sulfate-polyacrylamide gel electrophoresis
TBST	Tris-buffered saline buffer with Tween-20
TMA	Tissue microarray
TRITC	Tetramethylrhodamine-isothiocyanate
