# Supplementary material for: Human Costars Family Protein ABRACL Modulates Actin Dynamics and Cell Migration and Associates with Tumorigenic Growth
Source: Int J Mol Sci. 2021 Feb 18;22(4):2037. doi: 10.3390/ijms22042037 (PMC7922284; doi:10.3390/ijms22042037)
Supplement: Supplementary file 1 [file ijms-22-02037-s001.pdf]

Supplementary Materials for

Human Costars family protein ABRACL modulates actin dynamics and cell migration and associates with tumorigenic growth

Bo-Yuan Hsiao [1], Chia-Hsin Chen [1], Ho-Yi Chi [1], Pei-Ru Yen [1], Ying-Zhen Yu [1], Chia-Hsin Lin [2], Te-Ling Pang [1], Wei-Chi Lin [1], Min-Lun Li[1], Yi-Chen Yeh [3], Teh-Ying Chou [1,3,4,5] and Mei-Yu Chen [1,2,5,*]

[1] Institute of Biochemistry and Molecular Biology, National Yang-Ming University, No.155, Sec. 2, Linong St., Beitou Dist., Taipei 11221, Taiwan; BYHsiao0916@gmail.com (B.-Y.H.); s7532853@gmail.com (C.-H.C.); joeinter3690@gmail.com (H.-Y.C.); ruby781027@gmail.com (P.-R.Y.); clareyu77@gmail.com (Y.-Z.Y.); chlin1016@cgmh.org.tw (C.-H.L.); celine.pang@uwell.com.tw (T.-L.P.); weichi0905638@gmail.com (W.-C.L.); lazystream@gmail.com (M.-L.L.); tychou@vghtpe.gov.tw (T.-Y.C.); meychen@ym.edu.tw (M.-Y.C.)

[2] School of Medicine, National Yang-Ming University, No.155, Sec. 2, Linong St., Beitou Dist., Taipei 11221, Taiwan; chlin1016@cgmh.org.tw (C.-H.L.); meychen@ym.edu.tw (M.-Y.C.)

[3] Department of Pathology and Laboratory Medicine, Taipei Veterans General Hospital, No.201, Sec. 2, Shipai Rd., Beitou Dist., Taipei 11217, Taiwan; ycyeh2@vghtpe.gov.tw (Y.-C.Y.); tychou@vghtpe.gov.tw (T.-Y.C.)

[4] Institute of Clinical Medicine, National Yang-Ming University, No.155, Sec. 2, Linong St., Beitou Dist., Taipei 11221, Taiwan; tychou@vghtpe.gov.tw (T.-Y.C.)

[5] Cancer Progression Research Center, National Yang-Ming University, No.155, Sec. 2, Linong St., Beitou Dist., Taipei 11221, Taiwan; tychou@vghtpe.gov.tw (T.-Y.C.); meychen@ym.edu.tw (M.-Y.C.)

* Correspondence: meychen@ym.edu.tw; Tel.: +886-02-2826-7269

This file contains:

Supplementary Tables

Table S1. Plasmids and primers used in this study

Supplementary Figures and legends

Figure S1. ABRACL expression in human cancer cell lines

Figure S2. *In vitro* F-actin co-sedimentation assay

Figure S3. Testing ABRACL and cofilin in *in vitro* F-actin co-sedimentation

Figure S4. Upregulation of the *ABRACL* transcript level in various types of cancer

Supplementary Tables

Table S1. Plasmids and primers used in this study

Plasmid	Plasmid backbone	Primers for construction / Target sequences (5′--> 3′)	Description	Source
For expression of ABRACL in cancer cell lines				
pcDNA-HA-ABRACL	pcDNA3.0-HA	Forward primer: ACGGATCCATGAATGTGGATCACGA<br>Reverse primer: ACGAATTCTTAATCTTGCAGTAATATAATG	A PCR-amplified fragment containing the coding sequence (CDS) of *ABRACL* was digested with restriction enzymes (*BamH*I and *Eco*RI) and inserted into the pcDNA3.0-HA vector.	This study
pEGFP-C1-ABRACL	pEGFP-C1	Forward primer: ATCTCGAGCCATGAATGTGGATCACGAGGT<br>Reverse primer: GAATTCGCAGATCGTCAGTCAGTCAC	A PCR-amplified fragment containing the CDS of *ABRACL* was digested with restriction enzymes (*Xho*I and *Eco*RI) and inserted into the pEGFP-C1 vector.	This study
pcDNA3.1-myc-His-ABRACL	pcDNA3.1-myc-His(-)B	Forward primer: ACGACTCGAGCGATGAATGTGGATCACGAGGTTAAC<br>Reverse primer: AGGAAGCTTGGATCTTGCAGTAATATAATGTCAAC	A PCR-amplified fragment containing the CDS of *ABRACL* was digested with restriction enzymes (XhoI and *Hind*III) and inserted into the pCDNA3.1-myc-His(-)B vector.	This study
For Lentivirus-delivered shRNA-mediated gene knockdown				
pLKO-shLuc	pLKO_TRC005	Target sequence: GCGGTTGCCAAGAGGTTCCAT	Targeting the 3′-UTR of Luciferase gene	Academia Sinica, Taiwan
pLKO-sh295	pLKO_TRC005	Target sequence: TCCTCTTCCGTGATGATAAAT	Targeting the CDS of *ABRACL* gene	Academia Sinica, Taiwan
pLKO-sh484	pLKO_TRC005	Target sequence: TTCTGGTAAACTGGAATATAA	Targeting the 3′-UTR of *ABRACL* gene	Academia Sinica, Taiwan
pLKO-shCFL1	pLKO.1	Target sequence: CTATGAGACCAAGGAGAGCAA	Targeting the CDS of *CFL1* gene	Academia Sinica, Taiwan
For CRISPR/Cas9-mediated *ABRACL* knockout				
pgRNA-ABRACL-g1	gRNA_Cloning Vector	Forward primer: TTTCTTGGCTTTATATATCTTGTGGAAAGGACGAAACACCGTGGAGGAAATTCATCGTTT<br>Reverse primer: GACTAGCCTTATTTTAACTTGCTATTTCTAGCTCTAAAACAAACGATGAATTTCCTCCAC<br>Target sequence: TGGAGGAAATTCATCGTTT	Primers carrying a guide sequence for CRISPR/Cas9-mediated gene knockout were annealed and extended into a dsDNA fragment using the KOD plus polymerase. The 100-bp DNA fragment was then incorporated into the *Afl*II-linearized gRNA_Cloning Vector by the Gibson Assembly reaction.	This study
pgRNA-ABRACL-g2	gRNA_Cloning Vector	Forward primer: TTTCTTGGCTTTATATATCTTGTGGAAAGGACGAAACACCGCGAGGTTAACCTCTTAGTGG<br>Reverse primer: GACTAGCCTTATTTTAACTTGCTATTTCTAGCTCTAAAACCCACTAAGAGGTTAACCTCGC<br>Target sequence: CGAGGTTAACCTCTTAGTGG		This study

pgRNA-ABRACL-g3	gRNA_Cloning Vector	Forward primer: TTTCTTGGCTTTATATATCTTGTG GAAAGGACGAAACACCGATGA ATTTCCTCCACTAAG Reverse primer: GACTAGCCTTATTTTAACTTGCTA TTTCTAGCTCTAAAACCTTAGTG GAGGAAATTCATC Target sequence: ATGAATTTCCTCCACTAAG		This study
For expression of recombinant proteins in *E. coli*				
pGEX-5X-3-ABRACL	pGEX-5X-3	-	A fragment containing the CDS of *ABRACL* was obtained from pTX-GFP-mCostars[1] by *Eco*RI and *Xho*I digestion and inserted into pGEX-5X-3	This study
pRSET-C-CFL1	pRSET-C	Forward primer: ACGGATCCTTATGGCCTCCGGTG TGGCT Reverse primer: ACGAATTCTCACAAAGGCTTGC CCTCCA	A PCR-amplified fragment containing the CDS of the cofilin-1 gene *CFL1* was digested with restriction enzymes (*BamH*I and *Eco*RI) and inserted into pRSET-C.	This study
pRSET-C-CFL1-S3A	pRSET-C-CFL1	Forward primer: ATCGATGGATCCTTATGGCCGCC GGTGTGGCTG Reverse primer: GGCCATAAGGATCCATCGATCCT TATCGTC	Oligonucleotide-mediated site-directed mutagenesis was performed on pRSET-C-CFL1 using a PCR-based method.	This study
pRSET-C-CFL1-S3D	pRSET-C-CFL1	Forward primer: ATCGATGGATCCTTATGGCCGAC GGTGTGGCTG Reverse primer: GGCCATAAGGATCCATCGATCCT TATCGTC		This study
pRSET-C-CFL1-S3E	pRSET-C-CFL1	Forward primer: ATCGATGGATCCTTATGGCCGAA GGTGTGGCTG Reverse primer: GGCCATAAGGATCCATCGATCCT TATCGTC		This study

[1]Pang, T. L. et al. Costars, a *Dictyostelium* protein similar to the C-terminal domain of STARS, regulates the actin cytoskeleton and motility. *J Cell Sci* **123**, 3745-3755, doi:10.1242/jcs.064709 (2010).

Supplementary Figures and legends

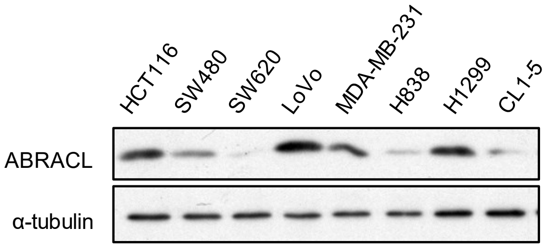

Figure S1. ABRACL expression in human cancer cell lines. Total cell lysates were collected and examined for the expression of ABRACL by Western analysis; α-tubulin expression was used as a loading control. HCT116, SW480, SW620, and LoVo are colon cancer cell lines. H838, H1299, and CL1-5 are lung cancer cell lines. MDA-MB-231 is a breast cancer cell line. All cell lines were obtained from the Bioresource Collection and Research Center, Taiwan.

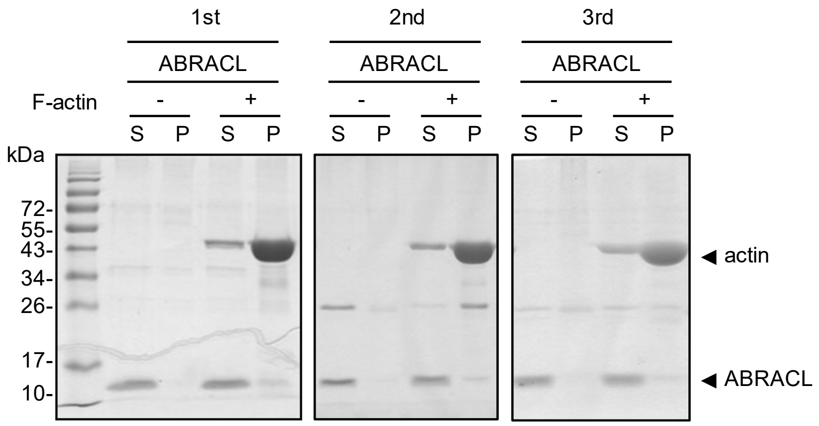

Figure S2. *In vitro* F-actin co-sedimentation assay. Recombinant ABRACL was purified as in Figure 4A and incubated with or without preformed F-actin. After centrifugation, supernatant (S) and pellet (P) fractions were analyzed by SDS-PAGE and stained with Coomassie Blue. Shown are three independent co-sedimentation tests performed as in Figure 4B. The gel image of the 3rd test is part of the image shown in Figure S3.

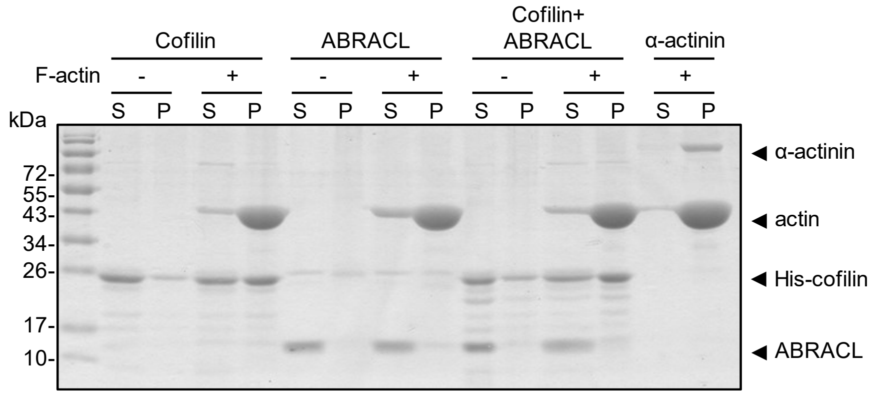

Figure S3. Testing ABRACL and cofilin in *in vitro* F-actin co-sedimentation. Reactions containing purified recombinant ABRACL (25 μM) and/or recombinant human His-cofilin (25 μM) incubated with or without preformed F-actin were subjected to centrifugation to sediment F-actin. Resulting supernatant (S) and pellet (P) fractions were analyzed by SDS-PAGE and stained with Coomassie Blue. Commercially available purified α-actinin was used as a positive control for F-actin binding.

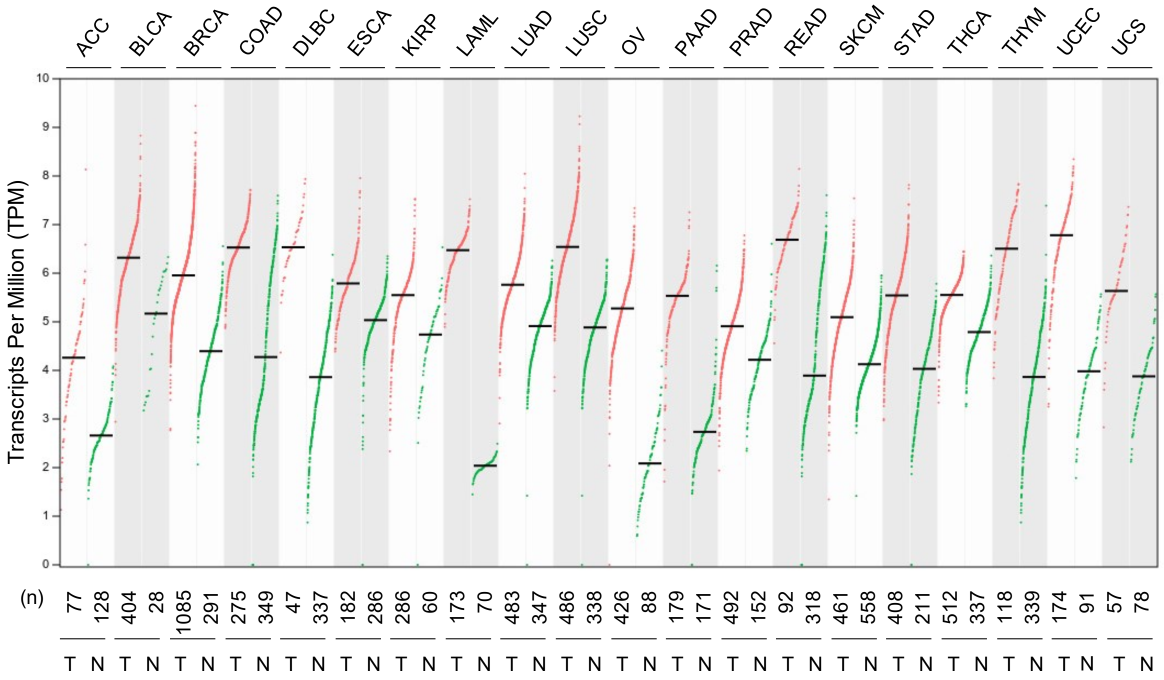

Figure S4. Upregulation of the *ABRACL* transcript level in various types of cancer. Shown are *ABRACL* transcript levels in corresponding tumor (T) and normal (N) tissues. The chart was derived using the GEPIA server, analyzing data from the Cancer Genome Atlas (TCGA) and Genotype-Tissue Expression (GTEx) databases. Cancer types are indicated on the top; ACC, adrenocortical carcinoma; BLCA, bladder urothelial carcinoma; BRCA, breast invasive carcinoma; COAD, colon adenocarcinoma; DLBC, lymphoid neoplasms diffuse large B-cell lymphoma; ESCA, esophageal carcinoma; KIRP, kidney renal papillary cell carcinoma; LAML, acute myeloid leukemia; LUAD, lung adenocarcinoma; LUSC, lung squamous cell carcinoma; OV, ovarian serous cystadenocarcinoma; PAAD, pancreatic adenocarcinoma; PRAD, prostate adenocarcinoma; READ, rectum adenocarcinoma; SKCM, skin cutaneous melanoma; STAD, stomach adenocarcinoma; THCA, thyroid carcinoma; THYM, thymoma; UCEC, uterine corpus endometrial carcinoma; UCS, uterine carcinosarcoma. Red and green dots represent data from cancerous and normal tissues, respectively; total numbers (n) of samples in each set are shown below the chart. Statistical analyses of the results indicated that *ABRACL* expression was significantly different ($p < 0.05$) between cancerous and normal samples in all types of cancer shown in this chart.
